# Evaluation of ChatGPT-4o in oral and maxillofacial surgery examinations: a comparative study of performance on U.S. dental decks and Chinese dental licensing examination practice questions

**DOI:** 10.1186/s12903-026-08551-9

**Published:** 2026-05-11

**Authors:** Hao Zheng, Zohreh Yousefian Zare, Menghong Li, Yuzhu Pan, Shuyao Ren, Haoyue Cui, Yuexiao Li

**Affiliations:** 1 School and Hospital of Stomatology, China Medical University, Shenyang, Liaoning 110002 China; 2 Department of Oral and Maxillofacial Surgery, School and Hospital of Stomatology, China Medical University, Shenyang, Liaoning 110002 China

**Keywords:** Artificial intelligence, Large language models, ChatGPT-4o, Education, Licensure, Dental

## Abstract

**Background:**

This study evaluates the performance of ChatGPT-4o on practice questions from the U.S. Integrated National Board Dental Examination (INBDE) and the Chinese Dental Licensing Examination to assess its potential and limitations in a high-stakes dental context.

**Methods:**

ChatGPT-4o and human participants (ten dental undergraduate students, ten graduate students specializing in oral and maxillofacial surgery (OMS), and ten OMS specialists with over five years of clinical experience) were tested on all available questions categorized under oral and maxillofacial surgery from the U.S. Dental Decks online platform (English) and *the 2024 Chinese Dental Licensing Exam Question Bank* (simplified Chinese). Accuracy (percentage of correct responses) was compared between groups using chi-squared tests.

**Results:**

ChatGPT-4o achieved an overall accuracy of 90% (*n* = 252) on the U.S. Dental Decks, outperforming all human participants on English-language questions (*p* < 0.001), with a moderate magnitude of difference. In contrast, its accuracy on the Chinese question bank was 71% (*n* = 567, *p* < 0.001 vs. English performance), a level comparable to that of dental undergraduate and OMS graduate students, but lower than that of OMS specialists (87%, *p* < 0.001), with differences of modest magnitude. By question type, accuracy on the U.S. Dental Decks was consistent across all formats. In the Chinese question bank, performance varied modestly by question type, with the highest accuracy observed for A1-type questions (76%) and the lowest for A2-type questions (61%).

**Conclusion:**

ChatGPT-4o demonstrated higher accuracy on English-language questions than on Chinese questions, suggesting a potential language-related performance gap. This finding highlights the importance of localized training data for Artificial Intelligence (AI) systems. Its performance in non-English contexts should be interpreted with caution, as it may not yet reach specialist-level expertise.

**Supplementary Information:**

The online version contains supplementary material available at https://doi.org/10.1186/s12903-026-08551-9.

## Background

The rapid advancement of artificial intelligence (AI), particularly large language models (LLMs) represented by Chat Generative Pre-trained Transformer (ChatGPT), has drawn widespread scientific interest and is increasingly integrated into medical practice and education [1–3]. ChatGPT is an AI chatbot developed by OpenAI and released in December 2022 [3]. Driven by machine learning algorithms, it generates text responses in response to user input. Leveraging its extensive training corpus, ChatGPT is capable of providing a broad range of medical information, thereby enhancing the autonomous learning of medical students [4, 5]. Its interactive, conversational interface reinforces understanding of medical concepts and supports deep learning. ChatGPT offers diverse functionalities for medical learning, including bilingual translation and quick reference retrieval, as well as serving as an interactive study assistant.

Medical licensing examinations are designed to assess the foundational knowledge and clinical competence of medical graduates, ensuring their readiness to provide safe and effective patient care [6, 7]. When preparing for medical exams, such as the United States Medical Licensing Examination (USMLE), ChatGPT can assist students by elaborating on disease classifications, analyzing data, clarifying complex physiological mechanisms, and even interpreting clinical scenarios or imaging findings [8]. Nevertheless, while generative AI tools like ChatGPT are quietly reshaping how medical students learn, the risk of misinformation in complex medical domains necessitates cautious and critical evaluation by educators [9]. One way to measure AI’s capabilities is by evaluating its accuracy in answering medical exam questions [10]. 

Although many studies have evaluated the scores of Large Language Models (LLMs) in dental licensing examinations [11–14], and significantly, Quah et al. [15]recently investigated the accuracy of various models specifically in oral and maxillofacial surgery (OMS), few studies have analyzed the performance gap between English and non-English systems. Furthermore, no study has specifically benchmarked the bilingual performance of the latest GPT-4o model against a full spectrum of human professional levels, ranging from dental undergraduates to OMS specialists.

Therefore, this study aimed to evaluate the performance of ChatGPT-4o on practice questions from the U.S. Integrated National Board Dental Examination (INBDE) and the Chinese Dental Licensing Examination, specifically focusing on OMS. The results could help determine its ability to accurately retrieve and apply dental knowledge, as well as explore its potential value in supporting dental education and personalized learning.

## Methods

### Ethical approval and consent to participate

Ethical approval was waived by the Medical Ethics Committee of the School of Stomatology, China Medical University, as the study posed minimal risk and involved anonymous, non-identifiable data collected for educational evaluation purposes only.

Informed consent to participate was waived by the Medical Ethics Committee of the School of Stomatology, China Medical University, as no personal identifiers were collected and participation did not affect academic evaluation or student welfare. All procedures were performed in accordance with the ethical standards laid down in the 1964 Declaration of Helsinki and its later amendments.

### Study materials

This study was conducted and reported in accordance with the Chatbot Assessment Reporting Tool (CHART) guidelines [16] to improve methodological transparency and reproducibility in artificial intelligence evaluation. This prospective study involved ChatGPT-4o and human participants from School and Hospital of Stomatology, who were tested on all OMS-related questions from the Dental Decks online platform and *the 2024 Chinese Dental Licensing Exam Question Bank*. ChatGPT-4o (OpenAI), released in May 2024, is a proprietary closed-source large language model. It was used as a pre-trained base model without additional fine-tuning in this study. A new ChatGPT Plus account was created at the beginning of the study to ensure a clean interaction history and avoid prior conversational influence.

### Participants

Human participants were recruited from a pool of eligible candidates at the School and Hospital of Stomatology, China Medical University. To establish a tiered professional benchmark, participants were divided into three cohorts (*n* = 10 per group): fifth-year dental undergraduate students who had completed all theoretical coursework, OMS graduate students, and OMS specialists. The OMS specialists were attending physicians with more than five years of active clinical experience in oral and maxillofacial surgery, who are concurrently engaged in clinical teaching and residency training at the School and Hospital of Stomatology. Given the exploratory nature of this study, a pragmatic sample size of 10 participants per group was adopted to provide an initial comparative benchmark. Participants were not financially compensated for their participation.

Inclusion and Exclusion Criteria: Participants were selected according to predefined criteria to ensure the reliability of the human benchmark. Inclusion required that all participants be native Chinese speakers and have passed the College English Test Band 6 (CET-6), providing a standardized level of English proficiency sufficient to understand the U.S. Dental Decks. Participants were excluded if they had prior exposure to or experience with the 2024 editions of either the U.S. Dental Decks or *the Chinese Dental Licensing Exam Question Bank*, as assessed by self-report during the recruitment process.

### Procedures and data collection

**Fig. 1:**
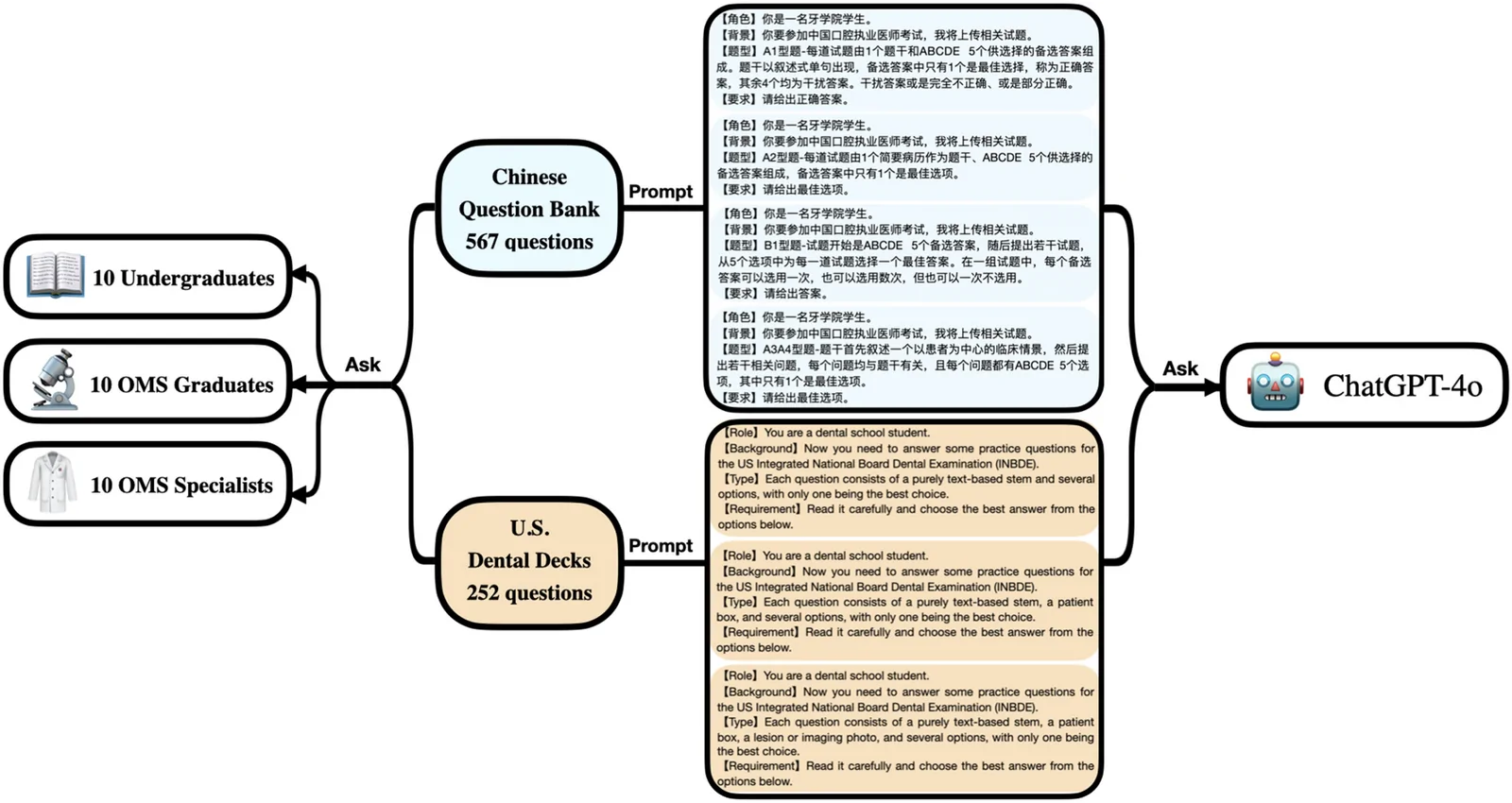
Workflow of the question-answering process and prompts provided to ChatGPT-4o. The study utilized 567 questions from *the 2024 Chinese Dental Licensing Exam Question Bank* and 252 questions from the Dental Decks platform. These questions were administered to both ChatGPT-4o and three groups of human participants: ten dental undergraduates, ten oral and maxillofacial surgery (OMS) graduates, and ten OMS specialists. To simulate examination conditions for the artificial intelligence (AI), specific prompts were designed for different question types in either Chinese or English

ChatGPT-4o was accessed through the ChatGPT web interface. All queries were conducted in August 2024 in Shenyang, China. Examination questions were obtained from the U.S. Dental Decks platform and *the 2024 Chinese Dental Licensing Exam Question Bank*. To simulate real examination conditions, standardized instructions were used for each question type (Fig. 1), asking ChatGPT to select the correct answer from the provided options. Prompts were presented in the original language of the examination materials—English for the U.S. Dental Decks and simplified Chinese for the Chinese question bank—to ensure linguistic consistency. The investigators uploaded screenshots of the original questions directly into ChatGPT rather than manually transcribing the content. This approach minimized transcription errors and preserved the original formatting, particularly dental notation. Prompt design and query submission were conducted by the investigators, and no patients or members of the public were involved in prompt development.

Regarding the testing sequence, ChatGPT-4o was first evaluated on the entire U.S. Dental Decks question set, followed by the Chinese dental question bank. To minimize the potential for sequential bias, context contamination, or in-context learning, each question was input into a new, independent chat session (i.e., a zero-shot setting). Once a response was generated, the session was terminated and the interaction history was cleared before presenting the next question. This approach was adopted to reduce the likelihood that prior interactions could influence subsequent responses. No feedback, correction, or evaluation was provided to ChatGPT-4o during the interaction process, ensuring that each response was generated independently without external influence.

The INBDE is the standardized written licensing exam for dentists in the USA, designed to evaluate the foundational knowledge and clinical reasoning of dental students and international graduates in the U.S. The Dental Decks review materials (used for over 30 years) remain one of the most popular and reputable resources for INBDE preparation.

In this study, all 252 questions were selected from the Oral Surgery section of the Dental Decks online platform (https://dentaldecks.com). The questions covered the following subtopics: Anesthesia (*n* = 38), Exodontia (*n* = 24), Fractures (*n* = 11), Grafts (*n* = 5), Implants (*n* = 4), Temporomandibular Joint (*n* = 4), Nerve Disorders (*n* = 5), Salivary Gland Disease (*n* = 16), Cysts, Tumors and Lesions (*n* = 61), Adrenal Cortex (*n* = 4), Drugs (*n* = 7), Anatomy (*n* = 24), Biopsy (*n* = 3), Disorders/Conditions (*n* = 13), General Information (*n* = 10), and Miscellaneous (*n* = 23). All questions were multiple-choice, consisting of 205 plain-text questions, 41 case-box questions, and 6 image-based questions.

The Chinese questions was derived from *the 2024 Chinese Dental Licensing Exam Question Bank* published by the People’s Medical Publishing House (PMPH). A total of 567 questions from the OMS module were included, covering the following subtopics: Basic Knowledge (*n* = 55), Anesthesia (*n* = 52), Exodontia (*n* = 69), Implants (*n* = 10), Infections (*n* = 65), Fractures (*n* = 61), Cysts, Tumors and Lesions (*n* = 101), Salivary Gland Disease (*n* = 35), Temporomandibular Joint (*n* = 41), Nerve Disorders (*n* = 20), Cleft Lip and Palate (*n* = 33), Dentofacial Deformities (*n* = 8), and Grafts (*n* = 17). The question types followed the official exam formats (A1, A2, A3, A4, and B1), representing a comprehensive and authoritative collection for Chinese dental board exam preparation.

It is important to note that all available OMS-related questions from both datasets were comprehensively included in this study, rather than being selected randomly or based on specific training priorities. A detailed side-by-side comparison of the question selection and subtopic allocation between the U.S. Dental Decks and the Chinese Question Bank is provided in Supplementary Table 1. To clarify the Chinese examination nomenclature for an international audience, the formats are defined as follows: (1) A1 (Single-statement best-answer): Consists of a single narrative sentence as the stem and five options (A-E), with only one correct best answer. (2) A2 (Case summary best-answer): Features a brief clinical case history as the stem, followed by five options with one best answer. (3) A3/A4 (Clinical scenario-based): Presents a comprehensive, patient-centered clinical scenario stem, followed by several related questions. Each question has five options with one best answer. (4) B1 (Extended matching items): Presents a shared pool of five options (A-E) first, followed by several related questions. For each question, the single best option must be selected; options may be used once, multiple times, or not at all.

It is important to note that ChatGPT did not interact with actual, secure exam questions from U.S. or Chinese licensing boards, as these are confidential and inaccessible to outside researchers. Consistent with prior research, publicly available or commercially published practice questions were used that approximate, but do not replicate, the real examination content. The questions were used for research purposes.

To compare ChatGPT-4o’s performance with that of human participants, the same sets of questions were compiled into formal examination papers and administered under standardized conditions, including a closed-book and timed format. The time limits were based on the Sino-US dental licensing examinations, averaging 48 s per Chinese question and 75 s per English question. To reduce cognitive fatigue, participants completed the two examinations on separate days, with a two-day interval between sessions. All participants completed the examinations in the same sequence: the Chinese question bank first, followed by the U.S. Dental Decks. Both testing sessions were conducted in a controlled environment (a quiet classroom without internet access, with no reference materials or peer discussion allowed). Regarding the response format, all questions followed the original multiple-choice format, with participants selecting a single best answer from the provided options. The language of the test materials was preserved in its original form—English for the U.S. Dental Decks and simplified Chinese for the Chinese dental question bank—to ensure consistency across test conditions. This design allows for the evaluation of ChatGPT-4o’s performance across different linguistic contexts.

### Outcomes and statistical analysis

The correct answers provided in the U.S. Dental Decks platform and *the 2024 Chinese Dental Licensing Exam Question Bank* were used as the reference standard. Responses from both ChatGPT-4o and human participants were compared with these predefined answer keys to determine accuracy. Given the deterministic nature of multiple-choice questions with a single correct answer, evaluator blinding was not implemented, as outcome assessment was objective and not subject to subjective interpretation. Two investigators independently verified the responses. Accuracy (percentage of correct responses) was used as the primary outcome. Categorical variables between two groups were compared using the chi-squared test. For multiple-group comparisons, omnibus chi-squared tests were performed first to assess overall differences, followed by Bonferroni-corrected pairwise chi-squared tests. Effect sizes were calculated using Cramer’s *V* to assess the strength of associations. *P*-values < 0.05 were considered statistically significant. All statistical analyses were conducted using SPSS 30.0 (IBM, Armonk, NY, USA).

## Results

Incorrect responses occurred when ChatGPT selected an option different from the reference answer. The study did not specifically evaluate harmful or biased responses, as the tasks involved answering standardized examination questions.

### Overall performance of ChatGPT-4o

**Fig. 2:**
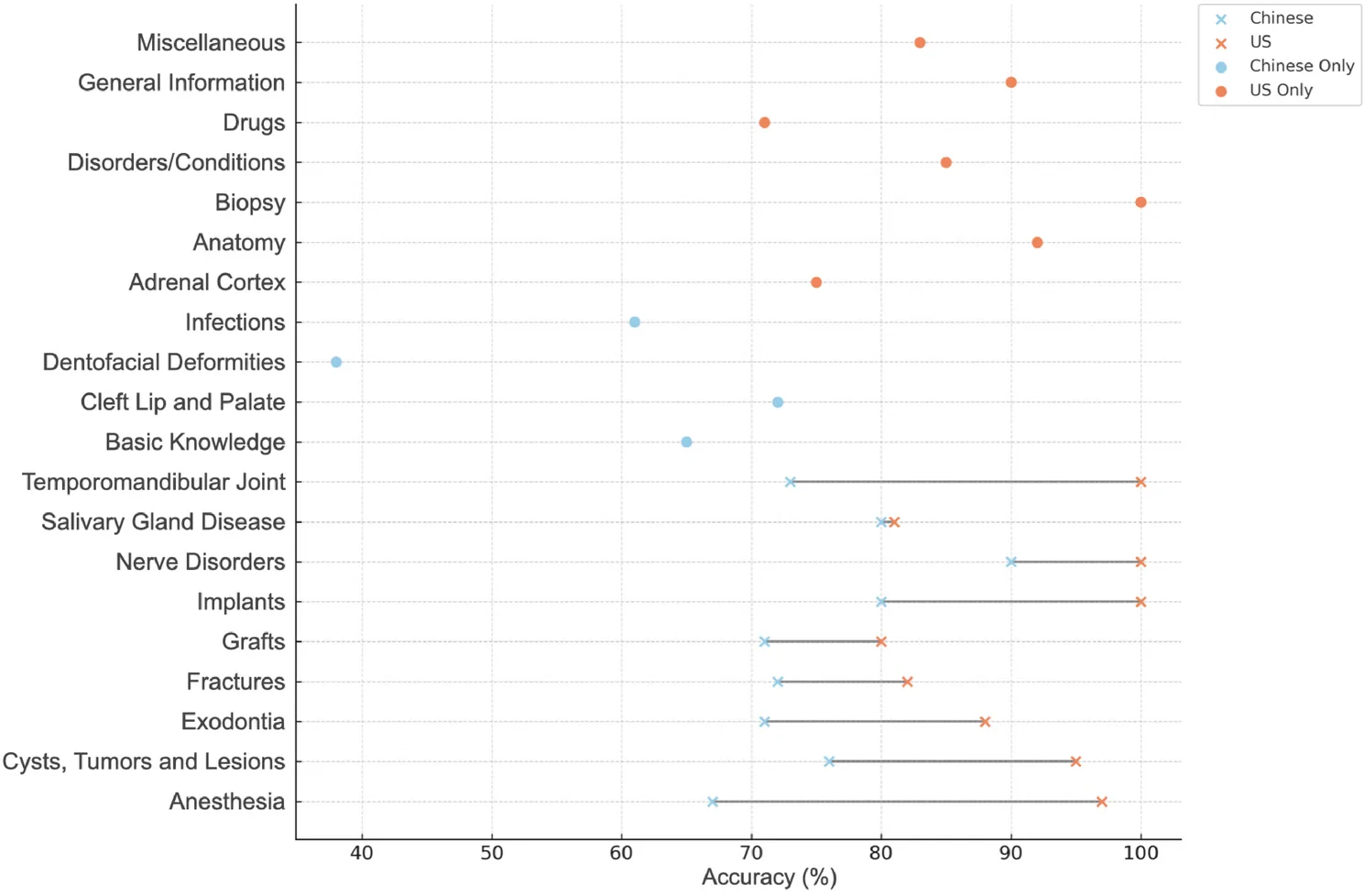
Subtopic-level performance of ChatGPT-4o on Chinese and U.S. dental licensing practice questions. Shared subtopics are visualized using dumbbell plots, where each line connects ChatGPT-4o’s accuracy on the Chinese question bank (left) and U.S. Dental Decks (right), enabling direct comparison within overlapping domains. Subtopics unique to a single dataset are shown as individual points to highlight differences in content coverage between the two question banks

In the Chinese question bank, ChatGPT-4o achieved an overall accuracy rate of 71% (*n* = 567). As shown in Fig. 2, the model performed particularly well in the following subtopics: Nerve Disorders (90%), Implants (80%), Salivary Gland Disease (80%), Cysts, Tumors and Lesions (76%). The lowest accuracy was observed in Dentofacial Deformities (38%), indicating that it is the only subtopic with an accuracy below 60%. In contrast, ChatGPT-4o demonstrated superior performance on the U.S. Dental Decks, with an overall accuracy rate of 90% (*n* = 252). The model achieved perfect scores (100%) in several domains, including Implants, Temporomandibular Joint, Nerve Disorders, and Biopsy. The lowest accuracy was observed in Drugs, at 71%.

### Comparison of performance between ChatGPT-4o and human participants

**Fig. 3:**
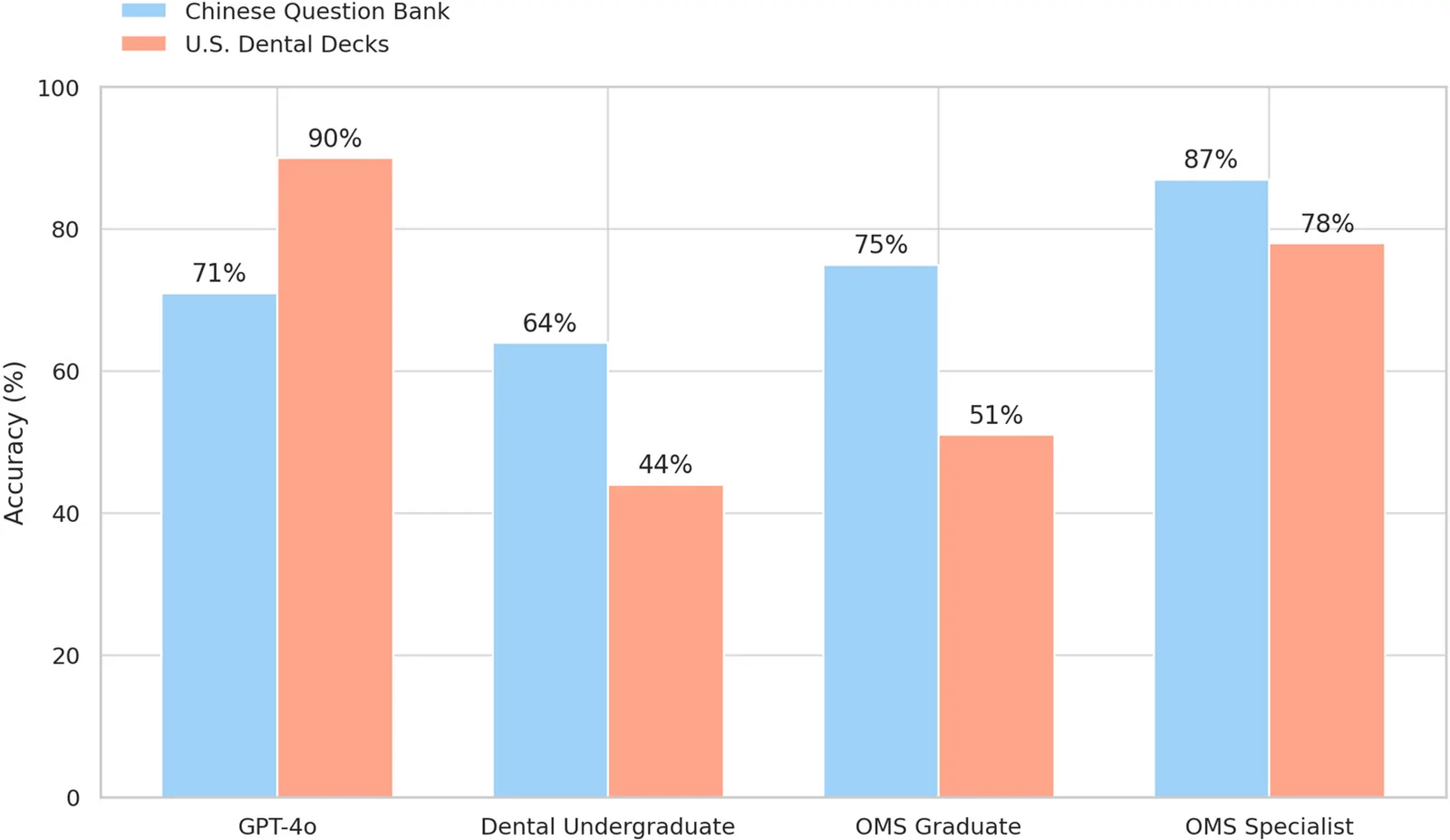
Performance comparison between ChatGPT-4o and human participants on Chinese and U.S. dental licensing practice questions. The chart compares the accuracy of ChatGPT-4o with that of dental undergraduates, oral and maxillofacial surgery (OMS) graduates, and OMS specialists. ChatGPT-4o’s accuracy on the Chinese questions (71%) was lower than that of OMS specialists (87%) but comparable to dental undergraduates (64%) and OMS graduates (75%). On the English questions, ChatGPT-4o (90%) outperformed all human groups

ChatGPT-4o’s performance differed markedly from that of human participants (dental undergraduate students, OMS graduate students, and OMS specialists) across both question sets (Fig. 3). As shown in Table 1, the four groups showed significantly different accuracy levels on the Chinese question set (*n* = 567) (χ² = 118.275, *p* < 0.001), with a small-to-moderate effect size (Cramer’s *V* = 0.228). The post hoc analysis (Bonferroni-adjusted chi-squared tests) revealed that ChatGPT-4o’s accuracy (71%) was significantly lower than that of specialists (87%), but not significantly different from that of dental undergraduates (64%) or OMS graduate students (75%).

**Table 1:** Performance comparison between ChatGPT-4o and humans on dental licensing practice questions in China and the United States

	Chinese Question Bank		U.S. Dental Decks		χ^2^	Cramer’s V	*p*
	(*n* = 567)		(*n* = 252)				
	Correct Answers	Accuracy	Correct Answers	Accuracy			
GPT-4o	403^ab^	71%	227^d^	90%	35.492	0.208	< 0.001
Dental undergraduate	364^b^	64%	111^e^	44%	29.078	0.188	< 0.001
OMS Graduate	425^a^	75%	129^e^	51%	45.02	0.234	< 0.001
OMS Specialist	516^c^	87%	197^f^	78%	25.49	0.176	< 0.001
*χ* ^2^	118.275		160.21				
Cramer’s *V*	0.228		0.399				
*p*	< 0.001		< 0.001				

Groups sharing the same letter are not significantly different. Cramer’s *V* was used to estimate effect size, with values of 0.1, 0.3, and 0.5 indicating small, medium, and large effects, respectively

Different superscript letters (a, b, c, d, e, f) indicate significant differences between groups (Bonferroni-adjusted pairwise comparisons, *p* < 0.05)

On the English Dental Decks questions (*n* = 252), ChatGPT-4o achieved a markedly higher accuracy rate of 90%, significantly outperforming all three human groups (χ² = 160.210, *p* < 0.001, Table 1), with a moderate effect size (Cramer’s *V* = 0.399), indicating a stronger association than that observed in the Chinese dataset. Interestingly, while ChatGPT-4o performed better on English questions than on Chinese ones, the human participants (across all three experience levels) performed better on the Chinese question set than on the English one.

### Accuracy analysis by question type

In the Chinese question set, the accuracy of ChatGPT-4o varied significantly by question type (χ² = 10.090, *p* = 0.018, Table 2), with a small effect size (Cramer’s *V* = 0.133). A1-type questions (single-statement best-answer format) had the highest accuracy at 76%, whereas A2-type questions (case summary best-answer format) showed the lowest accuracy at 61%. Pairwise comparisons revealed a statistically significant difference between A1 and A2 question types; no other inter-group differences were significant.

**Table 2:** ChatGPT-4o’s performance across different question types in the 2024 Chinese dental licensing exam question bank

Question Type	Total Questions	Correct Answers	Accuracy	χ^2^		Cramer’s V	*p*
A1	299	228^a^	76%	10.09	0.133		0.018
A2	110	67^b^	61%				
A3/A4	88	61^ab^	69%				
B1	70	47^ab^	67%				
Total	567	403	71%				

Groups sharing the same letter are not significantly different

Different superscript letters (a, b) indicate significant differences between groups (Bonferroni-adjusted pairwise comparisons, *p* < 0.05)

In contrast, ChatGPT-4o’s performance across three types of questions in the English set was relatively stable (Table 3): 90% accuracy on both plain-text and case-box questions, and 100% accuracy on image-based questions. The chi-squared test analysis showed no significant differences among question types (χ² = 0.686, *p* = 0.567). Although statistically significant differences were observed in some comparisons, the corresponding effect sizes were generally small to moderate, indicating that the magnitude of these differences was limited.

**Table 3:** ChatGPT-4o’s performance across different question types in the dental decks

Question Type	Total Questions	Correct Answers	Accuracy	χ^2^	Cramer’s V	*p*
Plain text	205	184	90%	0.686	0.052	0.567
Case box	41	37	90%			
Image	6	6	100%			
Total	252	227	90%			

## Discussion

ChatGPT is an LLM that demonstrated remarkable capabilities in various medical contexts, including in licensing exams, but its performance in dental licensing exams has not been thoroughly studied. This study evaluated the performance of ChatGPT-4o vs. humans on practice questions from the Chinese Dental Licensing Exam and the INBDE. The results demonstrate a difference in performance between the two language contexts: ChatGPT-4o achieved high accuracy (90%) on English OMS-related practice questions from the U.S. dental licensing exam, whereas its accuracy on the Chinese question set was lower (71%) and below that of OMS specialists. ChatGPT has the potential to be an effective educational tool in dental education, though further improvements are needed.

The performance disparity observed in this study highlights a critical challenge for global medical education: the risk of exacerbating educational inequality [17–19]. While ChatGPT-4o excels in English-language contexts, its significantly lower accuracy in Chinese suggests that AI-augmented learning is currently biased toward English-speaking populations. Crucially, this performance deficit is not merely an artifact of differing examination blueprints. As illustrated in Fig. 2, even within shared, overlapping OMS subtopics (such as Anesthesia, Cysts/Tumors, and Temporomandibular Joint), ChatGPT-4o consistently demonstrated substantially lower accuracy on the Chinese questions compared to the U.S. questions. This confirms that the barrier lies significantly in localized language comprehension and clinical reasoning, rather than purely in topic distribution. Rather than placing the onus solely on non-English speaking students to overcome language barriers, this finding emphasizes the urgent need to develop high-quality, localized medical corpora. If non-English resources are not actively improved, the integration of AI into dental education risks creating a ‘digital divide,’ where students in non-English systems are disadvantaged not by their medical aptitude, but by the linguistic limitations of the tools available to them.

From a clinical perspective, the significant gap between human experts and AI presents a serious patient safety warning [9, 20]. On the Chinese dataset, OMS specialists achieved an accuracy of 87%, markedly outperforming ChatGPT-4o at 71%. This 16% performance deficit demonstrates that the model is currently insufficiently reliable for clinical decision-making in the Chinese healthcare context [21]. Unlike in educational settings where errors can be corrected, reliance on such tools in clinical practice could lead to misdiagnosis or inappropriate treatment plans. Therefore, while ChatGPT-4o shows promise, it must be strictly limited to a supportive role and should not be trusted as an autonomous clinical decision support system until its performance parity with human specialists is established across all languages.

Finally, these results offer actionable insights for AI developers regarding the ‘English bias’ in training datasets [22]. The model’s specific failure on Chinese A2-type questions—clinical case summaries requiring higher-order clinical analysis and reasoning—where it scored only 61%, indicates a lack of deep contextual understanding rather than mere translation errors [23]. This suggests that the current architecture struggles to parse complex clinical narratives in non-English languages [19]. To bridge this gap, future model iterations must prioritize the inclusion of diverse, native-language medical case reports and reasoning chains in their training data, ensuring that the AI’s clinical reasoning capabilities are robust across different linguistic and medical systems.

Several limitations of this study should be acknowledged. First, the English and Chinese question sets were derived from two distinct examination systems with differences in structure, content distribution, and subtopic coverage. As a result, the datasets are not fully equivalent, which may limit direct comparability between the two assessments.

Although subtopic-level analyses were performed with a focus on overlapping domains, residual differences in content cannot be fully excluded. Second, the cognitive difficulty of questions across the two datasets was not formally assessed. Variations in the proportion of higher-order versus lower-order questions, as conceptualized by Bloom’s taxonomy, may have influenced the observed performance differences and should be considered when interpreting the results. Third, the human participant sample size was relatively small (*n* = 10 per group) and was designed for exploratory comparison rather than population-level inference. Although participants were selected using predefined inclusion and exclusion criteria to ensure baseline comparability, the findings may not be fully generalizable to broader populations. Fourth, prior exposure to the question banks was assessed based on self-report, which may be subject to recall bias or incomplete disclosure. Fifth, evaluators were not blinded to the source of responses, which may have introduced differential scrutiny; however, given the objective, single-answer nature of multiple-choice questions, the impact of this bias is likely minimal.

Finally, while efforts were made to minimize potential bias in AI evaluation through independent, zero-shot interactions, subtle effects related to prompt design or interaction conditions cannot be entirely ruled out. Despite these limitations, this study provides a pragmatic evaluation of ChatGPT-4o’s performance across different linguistic and educational contexts, offering insights into the potential impact of language on AI-assisted clinical reasoning.

## Conclusion

In conclusion, ChatGPT-4o demonstrated higher accuracy on English-language OMS-related questions than on Chinese questions. While its performance on English questions was comparable to that of highly trained participants, its accuracy in the Chinese context was lower and did not reach the level of OMS specialists. These findings suggest a language-related disparity in AI performance, highlighting the need for caution when applying such models in non-English clinical or educational settings. At present, ChatGPT-4o may be best used as a supplementary educational tool rather than an independent clinical decision-support system in non-English contexts. Future research should focus on improving localized medical datasets to enhance AI reliability across languages. 

## Supplementary Information


Supplementary Material 1.


## Data Availability

All data generated or analysed during this study are included in this published article.

## Abbreviations

(AI): Artificial intelligence
(LLMs): Large language models
(ChatGPT): Chat Generative Pre-trained Transformer
(OMS): Oral and maxillofacial surgery
(INBDE): Integrated National Board Dental Examination
