# Supplementary material for: Evaluation of ChatGPT-4o in oral and maxillofacial surgery examinations: a comparative study of performance on U.S. dental decks and chinese dental licensing examination practice questions
Source: BMC Oral Health. 2026 May 11;26:1247. doi: 10.1186/s12903-026-08551-9 (PMC13366629; doi:10.1186/s12903-026-08551-9)
Supplement: Supplementary file 1 — Supplementary Material 1. [file 12903_2026_8551_MOESM1_ESM.docx]

**Supplementary Table 1. Question Selection and Subtopic Allocation in U.S. Dental Decks and Chinese Dental Licensing Exam Question Bank**

| Subtopic | U.S. Dental Decks | Chinese Question Bank |
| --- | --- | --- |
| Miscellaneous | 23 | NA |
| General Information | 10 | NA |
| Drugs | 7 | NA |
| Disorders/Conditions | 13 | NA |
| Biopsy | 3 | NA |
| Anatomy | 24 | NA |
| Adrenal Cortex | 4 | NA |
| Infections | NA | 65 |
| Dentofacial Deformities | NA | 8 |
| Cleft Lip and Palate | NA | 33 |
| Basic Knowledge | NA | 55 |
| Temporomandibular Joint | 4 | 41 |
| Salivary Gland Disease | 16 | 35 |
| Nerve Disorders | 5 | 20 |
| Implants | 4 | 10 |
| Grafts | 5 | 17 |
| Fractures | 11 | 61 |
| Exodontia | 24 | 69 |
| Cysts, Tumors and Lesions | 61 | 101 |
| Anesthesia | 38 | 52 |
